# Idiopathic parietal bone thinning mimicking a bone lesion

**DOI:** 10.4314/gmj.v57i4.10

**Published:** 2023-12

**Authors:** Thomas Saliba, Alessandro De Leucio, Paolo Simoni

**Affiliations:** 1 Hopital Universitaire des Enfants Reine Fabiola (HUDERF), Brussels, Belgium

**Keywords:** Ventouse, ventouse-assisted birth, focal parietal thinning, skull thinning, parietal bone thinning

## Abstract

Idiopathic focal unilateral skull thinning is a rare finding. An explanation, such as trauma or disease, can often be found. However, in some cases, no explanation is forthcoming, and thus, we must look further into their history for a possible cause. The case of a nine-year-old boy with a crescent-shaped unilateral parietal bone thinning and a history of ventouse-assisted birth is presented. The lesion matches a ventouse's typical location, shape, and size. Thus, with the support of one other reported similar case, we hypothesise this may be the origin. We present the case of a crescent-shaped lesion matching the imprint of a ventouse in a child with a concordant history. This finding is put in the context of similar reports in the literature, and we believe that this report provides further evidence of this obscure phenomenon.

**Funding:**

None declared

## Introduction

Idiopathic focal skull thinning (IFST) is rare and often asymptomatic.^1^ It can, however, be associated with pain or masses and represents a diagnostic challenge due to the spectrum of differential diagnoses.^1^ To date, only two cases of IFST have been described in children, both patients having a history of ventouse-assisted delivery. 2 We report the case of unilateral IFST in a young boy with no relevant traumatic history but ventouse-assisted delivery.

## Case Report

**Figure 1:**
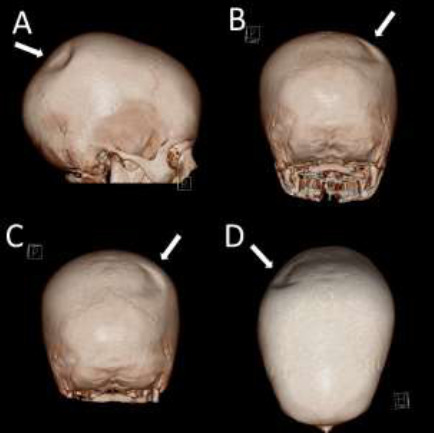
3D virtual reconstruction of the skull showing a crescent-shaped indentation on the posterior third of the right parietal bone of around 5cm in diameter (arrows). A) Right sided view. B) and C) Posterior views clearly demonstrating the crescent shaped indentation. D) Top-down view demonstrating the crescent shape and position of the indentation

**Figure 2:**
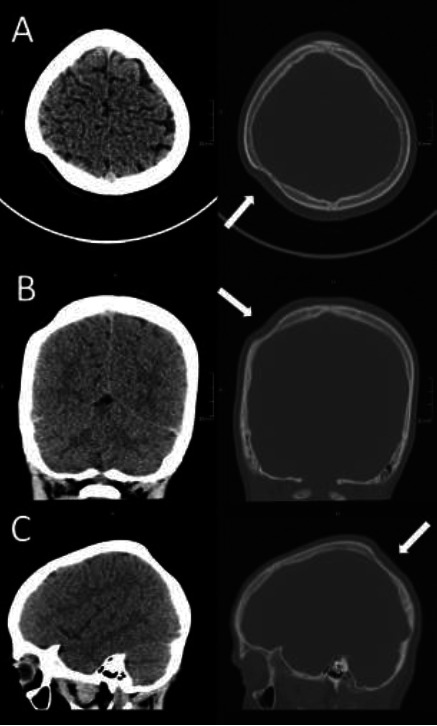
Non-contrast CT-scan in the 3 axes in the bone and tissue windows showing the thinning of the skull (arrows) and the normal appearance of the underlying brain tissue. Image A represents an axial view with tissue windowing (left image) and bone windowing (right image), image B represents a coronal view with tissue and bone windowing, and image C represents a sagittal view with tissue and bone windowing

A nine-year-old male presented to the emergency department for a severe headache centred around the left forehead and consisting of one to two episodes a day, with accompanying nausea. The patient reported that the pain increased during exercise but that it was relieved by orally administered paracetamol. The patient's history revealed head trauma to the left forehead ten days previously due to a fall whilst in physical education class, resulting in loss of consciousness and admission to the emergency department of a nearby hospital. The patient underwent a 4-hour observation period before being subsequently discharged. On the day of presentation to our emergency department, no visible scalp lesions were present upon clinical examination. A non-contrast head CT scan to rule out intracranial haemorrhage or skull fracture was ordered. The CT scan found signs of maxillary sinusitis but did not find signs of haemorrhage or fracture. However, a crescent-shaped thinning of the outer table of the right parietal bone approximately 5cm in diameter was discovered (Figure 1). The inner table of the skull and underlying brain tissue appeared normal (Figure 2).

The past medical history was negative for previous head trauma but revealed an assisted birth with no other known perinatal trauma. The patient's paediatrician was also contacted, providing no further information as to a traumatic event liable to have resulted in this lesion. After the CT scan, the patient returned to the emergency department. It was judged that he needed no further treatment, and was subsequently discharged home.

## Discussion

IFST is a rare disorder commonly presenting as bilateral and symmetrical parietal bone thinning, generally associated with elderly female patients. 3,4 The discovery tends to be incidental and thus is generally observed in patients undergoing head CT scans for other reasons.^3^ IFST can involve the inner and outer table of the bone and, in severe cases, perforates the dura.^3^,^4^ The aetiology of IFST is poorly understood, but some studies suggest the role of osteoporosis, particularly due to IFST's higher prevalence in post-menopausal women.^3^,^4^

Gorham-Stout disease (G-SD) is another possible differential diagnosis, presenting as progressive osteolysis with vascular and lymphatic proliferation.^5^ G-SD has only a hundred cases reported, affects both sexes at the same frequency and has an average age of diagnosis of 25.^5^ G-SD is of unknown aetiology but may be post-traumatic. 5 G-SD lesions generally cause pain, possibly with oedema, muscle weakness and location-dependent functional impairment, but can equally be asymptomatic.^5^ As no standard diagnosis guidelines exist, it is principally a clinical diagnosis of exclusion.^5^

CT-scans show osteolysis and, sometimes, vessel-shaped defects around the lesion's edges, with histology confirming the diagnosis.^5^ As our patient lacked osteolytic lesions, G-SD is unlikely.

Trauma is another possible differential diagnosis of IFST. However, the patient's skull didn't present any fracture line on CT examination. Furthermore, the remote pathological anamnesis was negative for skull trauma.

Watson et al. described two patients with focal crescent-shaped skull thinning who had also undergone ventouse-assisted births.^2^ In their work, the authors suggest the skull lesions may result from ventouse cup placement during delivery.^2^ The location just beside the centre of the skull convexity, a common ventouse location if it is not perfectly centred, as well as the size and round morphology of the lesions, further reinforce this theory.^6^

Although further evidence is required, a link between the ventouse placement during obstetric manoeuvres and the occurrence of skull thinning in children can be hypothesised. The strong similarity of the skull lesions between the three patients makes it a plausible explanation for the radiological findings.

Although other lesions also involve the skull, such as Langerhans cell histiocytosis, aplasia cutis congenita, and many metabolic and tumoral pathologies, none have similar characteristics to our patient's.^1^

**Ethical Considerations**: The patient images and data were fully anonymised. The patient's guardian provided informed consent to publish this case.

## Conclusion

Many causes for calvaria defects exist, ranging from iatrogenic to metabolic diseases, amongst many others. IFST is amongst the rarest aetiologies. With this report, we hope to make other medical practitioners aware of the existence of such lesions so that they might not be mistaken for a pathology.
